# An Efficient Image Deblurring Network with a Hybrid Architecture

**DOI:** 10.3390/s23167260

**Published:** 2023-08-18

**Authors:** Mingju Chen, Sihang Yi, Zhongxiao Lan, Zhengxu Duan

**Affiliations:** 1 School of Automation and Information Engineering, Sichuan University of Science & Engineering, Yibin 644002, China; 2 Artificial Intelligence Key Laboratory of Sichuan Province, Sichuan University of Science & Engineering, Yibin 644002, China

**Keywords:** image deblurring, hybrid architecture, transformer, cross-layer feature fusion

## Abstract

Blurring is one of the main degradation factors in image degradation, so image deblurring is of great interest as a fundamental problem in low-level computer vision. Because of the limited receptive field, traditional CNNs lack global fuzzy region modeling, and do not make full use of rich context information between features. Recently, a transformer-based neural network structure has performed well in natural language tasks, inspiring rapid development in the field of defuzzification. Therefore, in this paper, a hybrid architecture based on CNN and transformers is used for image deblurring. Specifically, we first extract the shallow features of the blurred images using a cross-layer feature fusion block that emphasizes the contextual information of each feature extraction layer. Secondly, an efficient transformer module for extracting deep features is designed, which fully aggregates feature information at medium and long distances using vertical and horizontal intra- and inter-strip attention layers, and a dual gating mechanism is used as a feedforward neural network, which effectively reduces redundant features. Finally, the cross-layer feature fusion block is used to complement the feature information to obtain the deblurred image. Extensive experimental results on publicly available benchmark datasets GoPro, HIDE, and the real dataset RealBlur show that the proposed method outperforms the current mainstream deblurring algorithms and recovers the edge contours and texture details of the images more clearly.

## 1. Introduction

Image deblurring is the task of reconstructing a high-quality clear image by removing unnecessary blur from a degraded input. Camera shaking caused by unstable hand holding while shooting, the rapid movement of objects, etc., are the main causes of image blurring, and can affect the quality and efficiency of the subsequent intelligent computer analysis of images. In the task of deblurring dynamic scene images, the resulting blur is non-uniform due to the ill-posed nature of the single image blur region, which greatly hinders this task.

Traditional image deblurring is usually performed using a natural image a priori due to its morbid development. Some methods [1,2] simplify this problem by assuming that the fuzziness is spatially invariant, which leads to a high time complexity of the solution process and does not correspond to the realism of dynamic scenarios. Some work [3,4,5] has been carried out on natural images by a priori modeling methods to remove non-uniform blur; however, a priori modeling methods have a limited ability to portray clear image features and non-uniform blur is difficult to simulate. In addition, sparse recovery is widely used for traditional image deblurring. Cai et al. [6] removed non-uniform blur by simultaneously maximizing the sparsity of the blurring kernel and the sparsity of the clear image. Zhang et al. [7] proposed an algorithmic framework for Bregmanized operator splitting (BOS), which solves the sparse recovery problem. Zhang et al. [8] proposed a blind image deblurring method based on sparse representation to remove non-uniform blur. Rostami et al. [9] applied the sparse recovery method of derivative compressed sensing (DCS) to solve the optical image deblurring problem. Yin et al. [10] used an l_0_ regular sparsity constraint-based learning method to solve the blind image deblurring problem. However, several works [6,7,8,9,10] suffer from drawbacks such as high computational complexity, dependence on a specific framing system, and sparsity assumptions made on images that cannot be applied to all images. Therefore, the traditional image deblurring methods have great limitations in solving the blur caused by complex factors.

In recent years, deep learning methods based on Convolutional Neural Networks (CNNs) have achieved remarkable results in the field of image deblurring, generally by building end-to-end network structures using CNN to learn feature mapping between blurred and clear images. Several studies have improved the deblurring effect by improving image deblurring methods based on recurrent structures, such as multi-scale (MS) [11,12], multi-patch (MP) [13,14], and multi-temporal (MT) [15]. However, in dynamic scenes, the blurring of images is usually regionally indeterminate and directionally different, and the degree of blurring varies, so a more efficient model to explore local and global correlations becomes an urgent problem.

Recently, thanks to the excellent performance of transformers [16] on natural language [17,18] and advanced vision tasks [19,20], the introduction of the transformers’ architecture to computer vision tasks has also gained a bright future. Image restoration tasks (e.g., denoising, defogging, rain removal) have also benefited from this, but few more suitable and efficient transformers models have emerged in the field of image deblurring. The main reason is that the computational complexity of the core self-attention mechanism (SA) grows quadratically with the increase in local pixels in high resolution images.

To address the above problem, a hybrid residual encoder–decoder architecture [21] consisting of CNN and a transformer is constructed in this paper to achieve image deblurring. Inspired by [22,23,24], an efficient stripe-based image deblurring transformer block with local and global connectivity is modeled, which we have called ESIDformer. Specifically, it is a self-attention mechanism that attaches in different directions when dealing with fuzzy regions of different sizes, using intra- and inter-strip attention to reorganize fuzzy features. Intra-strip attention formed for intra-strip markers carries local pixel blurring information, while inter-strip attention formed for inter-strip markers expresses global pixel blurring information. The interlocking stacked intra- and inter-strip attention layers’ interactions carry a wealth of ambiguous information. Meanwhile, in order to fully fuse shallow feature information, we have designed cross-layer feature Fusion Blocks. In addition, to achieve efficient feature transformation, we employed dual gating feedforward networks (DGFN) and deep convolution for local information enhancement in feedforward neural networks. Finally, the clear image after deblurring was recovered via convolution and a cross-layer feature fusion block (CFFB).

In summary, the main contributions of the work in this paper are as follows:

- We propose the ESIDformer network, a hybrid residual encoder–decoder architecture consisting of CNN and a transformer, for a multiscale local and global training framework for image deblurring. Based on the structure of a standard transformer, a strip-based transformer block (STB) is constructed, which can construct intra- and inter-strip tokens and stack them interleaved so as to more closely aggregate local and global pixels, and effectively handle blurred-clear image pairs.
- A dual gating feedforward network (DGFN) is designed, which fuses useful information in both paths of the element, thus achieving the effect of enriching the larger end of the information and suppressing the smaller end of the information.
- A cross-layer feature fusion block (CFFB) is designed, which adaptively fuses layered features with learnable correlations between different layers.
- We demonstrate the validity of our method by demonstrating it on the benchmark datasets GoPro, HIDE, and the real dataset RealBlur. In addition, we provide quantitative results, qualitative results, and results of ablation experiments.

## 2. Related Work

### 2.1. CNN-Based Image Deblurring Architecture

The purpose of image deblurring is to remove blurring artifacts from a degraded image to restore a clear image. CNN-based architectures have achieved impressive results compared to traditional algorithms, and most of these approaches are based on recurrent constructs to improve model performance. For example, Nah et al. [12], inspired by the traditional coarse-to-fine method, used a multi-scale convolutional neural network to blindly deblur images and a multi-scale loss function to constrain the network training process. Further derived from the above approach, Tao et al. [25] proposed a scale recurrent network structure to solve the image deblurring problem in dynamic scenes. Zhang et al. [26] proposed a spatially transformed recurrent neural network to solve the deblurring problem in dynamic scenes. A large number of end-to-end deep learning methods have been proposed successively for the deblurring problem in dynamic scenes [11,27,28]. In addition, based on the successful application of Generative Adversarial Networks in image restoration problems, Kupyn et al. [29,30] used conditional adversarial networks for deblurring. In recent years, Zamir et al. [31] have proposed a multi-stage architecture, where the entire deblurring process is decomposed into multi-stage recovery by learning the degraded inputs step by step. Cho et al. [32] proposed a multiple-input multiple-output U-Net (MIMO-UNet) for efficient deblurring.

### 2.2. Visual Attention Mechanism

As deep learning continues to advance in the field of image deblurring, more and more work is carried out considering the design of network structures in conjunction with non-uniform blurred image properties. Visual attention mechanisms are capable of locating target regions in images and capturing features of regions of interest, and have been successfully applied to recognition and classification problems. To address the feature of non-uniform blurred images differing in blurring degree and blurring type in different regions, literature [33] applied a visual attention mechanism to the image deblurring task and proposed an attention module and deformable convolution module for the dynamic scene deblurring task. Literature [34] proposed a simple and effective selective attention module, which is capable of adaptively recovering images by dynamically adjusting the weights of the operations according to the differences in the degradation level of different input images. A gated fusion CNN module has been proposed in the literature [35]. The method combines the characteristics of blurred images in real scenes and provides a feasible idea for subsequent work.

The self-attention mechanism and multi-head self-attention were then created. The former mimics the saliency detection and selective attention of biological vision, which can establish long-range dependencies and solve the problem of the limited perceptual field of CNN. The latter solves the problem of the single-head attention mechanism being limited by the feature space, as well as the lack of modeling capability.

### 2.3. Vision Transformer

A transformer was first applied to machine translation tasks in natural language processing (NLP) as a sequence-to-sequence autoregressive model. The transformer utilizes self-attention, cross-attention, and positional coding to produce capabilities that traditional CNNs do not possess, including a strong overall perception of images, the scalability of models, adaptability to multimodal data, and so on. The Vision Transformers (VIT) [36] model utilizes the classic transformer encoder structure to achieve the image classification task and is at the beginning of low-level visual transformer models. Specifically, it first converts the input images into non-overlapping, fixed-size image blocks, secondly flattens each image block into a one-dimensional vector, and then compresses the dimensionality via linear projection. In addition, learnable classification flag bits are introduced at the front end of the image sequence to enable the classification task, and then the position information is added using position coding and fed into multiple serial standard transformer encoders for attention computation and feature extraction. Influenced by VIT, the transformer is mushrooming in applications for underlying vision tasks. These include image generation [37,38], super-resolution image reconstruction [39,40], image enhancement [41], and image inpainting. Uformer [42] is a typical model of transformer for image deblurring. Uformer replaces the convolutional layer in U-Net with an encoder and a decoder. The encoder is responsible for extracting features of degraded images and the decoder is responsible for reconstructing images, both of which are designed as transformer modules with locally enhanced windows to capture long-range dependencies using a window-based non-overlapping self-attention mechanism, reducing the computational complexity of the model. However, the Uformer is limited by the 8 × 8 square window, which may result in the transformer not receiving enough contextual information, and the channel-based attention mechanism may lose some spatial information. In addition, the above methods do not combine convolution and attention mechanisms well, which limits the ability of the model to extract complex features.

To address the above issues, we extend the application of strip labeling, not only for intra-strip attention computation, but also for inter-strip attention computation, to better capture the patterns of fuzzy features. Refs. [23,43] used vertical and horizontal attention to capture global image dependencies. Inspired by them, we consider both horizontal and vertical pixel correlations and use a skip connection to make fuller use of the image information. This approach improves the model’s ability to understand and represent image-blurred regions using a priori blurred patterns, while reducing the number of tokens and parameters, and is more able to remove motion blur.

## 3. Approach

In this section, we first describe the overall network and hierarchical structure of the ESIDformer. Then, several core components of the ESIDformer are detailed, including the cross-layer feature fusion block (CFFB), strip-based multi-headed self-attentiveness (S-MSA), and the dual gating feedforward network (DGFN). Conditional positional encoding (CPE) was introduced after DGFN [44].

### 3.1. Network Architecture

**Figure 1:**
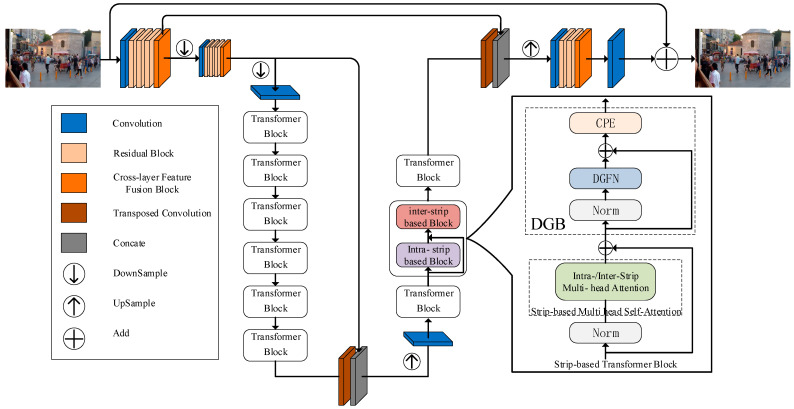
The overview of the proposed ESIDformer framework. The input is a blurred image and the output is a deblurred image.

Figure 1 illustrates the overall model design of the ESIDformer, a residual encoder–decoder architecture consisting of CNN and transformer, designed to solve the problem of image blurring in dynamic scenes. Specifically, the input-blurred image is first passed through two shallow feature embedding blocks (SFEB), and the shallow features are extracted by downsampling through the projection layer, and the resolution is one-fourth of the input after two SFEBs. Immediately after, interleaved, stacked intra- and inter-strip transformer blocks are used at the smallest and second-smallest scales to capture blur information of the different orientations and sizes at different scales in the image. Subsequently, transposed convolution is taken for upsampling, and its output features are concatenated with those generated by the encoder at the same scale. The depth features generated by the decoder processing are then transposed and convolved to recover to the resolution size at input, followed by two residual blocks and a cross-layer feature fusion block to generate the enhanced features for image deblurring. Finally, the ESIDformer performs 3×3 convolution of the output features to obtain the deblurred image.

### 3.2. Shallow Feature Embedding Block

In general, the vanilla transformer splits the input image into many image blocks, which are processed independently in the transformer [36,41]. As the features in the image blocks are stretched into one-dimensional vectors as input, this leads to a squeezing of correlation in the pixel space, resulting in a loss of feature information and a rapid growth of parameters that subsequently capture long-range dependencies. There are also some models that use feature connectivity or skip connectivity to avoid the loss of feature information [42,45]. However, these approaches do not fully explore the association between different layers, thus limiting the expressiveness of the input features.

**Figure 2:**
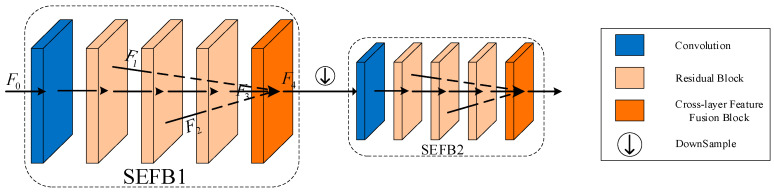
The architecture of shallow feature embedding blocks. $F_{0}$ is the input feature, $F_{1}$, $F_{2}$, and $F_{3}$ are the extracted features of the residual block, and $F_{4}$ is the fused feature.

**Figure 3:**
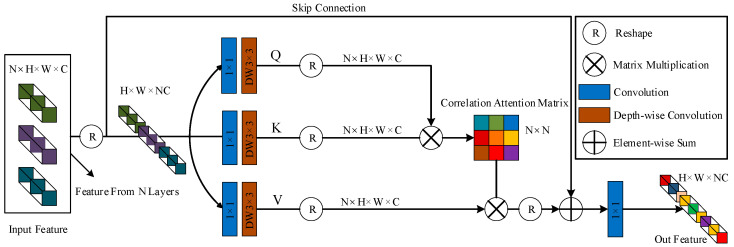
Detailed structure of the cross-layer feature fusion block. It corresponds to the orange block in Figure 2.

To address the above problem, we use two shallow feature embedding blocks (SFEB), each consisting of a convolutional layer, three residual blocks, and a cross-layer feature fusion block (CFFB) (as shown in Figure 2), to generate feature embeddings. This effectively avoids the loss of spatial information and reduces the number of parameters required. In addition, the CFFB allows for a better interaction of feature information between different layers. Specifically, the intermediate features outputs from the three residual blocks are denoted as $F_{1},\;F_{2},\;F_{3}\in R^{H\times W\times C}$, where H, W, and C denote the height, width, and number of channels, respectively. $F_{1}$, $F_{2}$, $F_{3}$ are aggregated and transformed into the enhanced features $F_{4}$ by the proposed cross-layer feature fusion block. We designed the CFFB as a layered feature that adaptively fuses different layers with learnable correlations between them. The CFFB architecture is shown in Figure 3. Given a connected feature $F_{i}\in R^{N}{}^{\times H\times W\times C}$ with N consecutive layers (N=3 in this paper), we first extend $F_{i}$ to generate an ${\hat{F}}_{i}$ with dimensions H×W×NC. Then, a 1×1 convolutional layer is used to mix the dependencies across channel contexts and a 3×3 deep convolution is used to encode the spatial feature information on the channels, denoted as Q, K, and V. Next, the queries and keywords are tensor-reshaped to obtain the two-dimensional matrices of $N\times HWC\;(\hat{Q})$ and $HWC\times N\;(\hat{K})$, and the N×N correlation attention matrix is calculated. Finally, the reshaped $\hat{V}\in R^{HWC\times N}$ is multiplied by the proportional correlation factor α to obtain the correlation attention matrix Y, and concatenated with $F_{i}$. Overall, the CFFB process is formulated as:

(1) $$\begin{array}{l} {\hat{F}}_{o}=W_{1\times 1}CFFB\_Attention\;(\hat{Q},\hat{K},\hat{V})+{\hat{F}}_{i}\\ CFFB\_Attention(\hat{Q},\hat{K},\hat{V})=\hat{V}softmax(\hat{Q}\cdot \hat{K}/\alpha ) \end{array},$$

where ${\hat{F}}_{0}$ is the mapping of the output features. In the overall network structure, we place the proposed CFFB after the residual blocks to emphasize the local context and fuse the features of different layers more effectively during feature extraction and image deblurring.

### 3.3. Stripe-Based Transformer Block

The transformer is ahead of CNN in modeling non-local self-similarity and remote dependencies. However, previous studies have shown that the computational cost of a standard transformer increases quadratically with the size of the space (H×W). To address the above problem, in Transformer Block we propose a stripe-based multi-head self-attention (S-MSA) mechanism. Since the complexity of the S-MSA calculation is linear in spatial size, it reduces the computational cost. In addition, we use a dual gating feedforward network (DGFN) instead of the conventional multilayer perceptron (MPL) to capture more important feature information. We have combined the above units to construct the strip-based transformer block (STB). As shown in Figure 1, an STB contains an S-MSA, a DGFN, and two normalization layers. The STB is described as:

(2) $$\begin{array}{l} {\hat{F}}_{i}=S-MSA\left(Norm\left(F_{i}\right)\right)+F_{i}\\ \begin{array}{l} {\hat{F}}_{o}=CPE\left(DGFN\left(Norm\left({\hat{F}}_{i}\right)\right)+{\hat{F}}_{i}\right)\\ F_{o}={\left({\hat{F}}_{o}\right)}_{Intra-SA}+{\left({\hat{F}}_{o}\right)}_{Inter-SA} \end{array} \end{array},$$

where $F_{i}$ is the input of STB, ${\hat{F}}_{i}$ and ${\hat{F}}_{o}$ are the outputs of S-MSA and DGFN, respectively, and ${\left({\hat{F}}_{o}\right)}_{Intra-SA}$ and ${\left({\hat{F}}_{o}\right)}_{Inter-SA}$ are the outputs of intra-strip attention and inter-strip attention, respectively. CPE is conditional positional encoding and Norm is layer normalization. It is important to note that $F_{i}$ represents the inputs, which can be either intra-stripe or inter-stripe. These inputs are interleaved and stacked together. Additionally, the inter-strip inputs receive outputs from the intra-strip as well as inputs from the intra-strip skip-connection, as illustrated in Figure 1. This design enables the comprehensive utilization of shallow features. In the following, we will provide detailed explanations of each module of STB individually.

An S-MSA block consists of an intra-SA block and an inter-SA block, which are interleaved and stacked in the network. We detail the functional roles of these two as follows.

#### 3.3.1. Intra-SA Block

**Figure 4:**
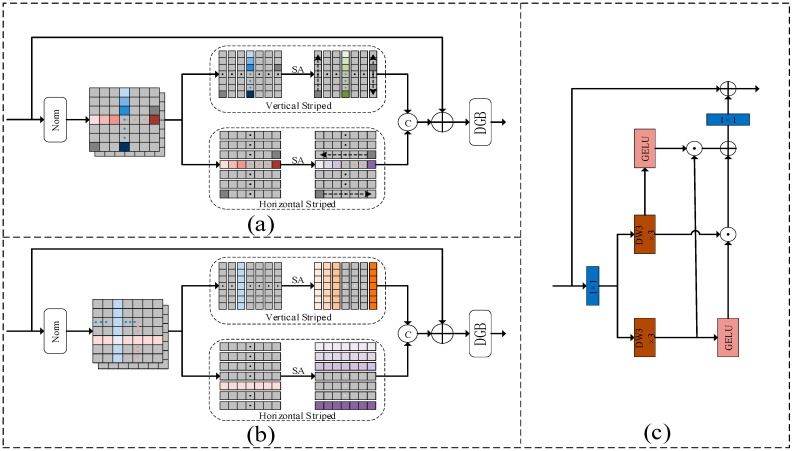
Here, (**a**) represents intra-SA long-term dependency modeling, (**b**) represents inter-SA long-term dependency modeling, where © denotes concatenation, and (**c**) represents dual gating feedforward network.

As shown in Figure 4a, the input features of an intra-SA block are divided into two independent features in parallel, vertical intra-strip attention (Intra-SA-V) and horizontal intra-strip attention (Intra-SA-H), and then they are subjected to SA operations separately. Let the input characteristics of the intra-strip block be $I\in R^{H\times W\times C}$, where H, W, and C denote the height, width, and number of channels, respectively. Considering that the vertical and horizontal strip notes are crossed, we first preprocess with LayerNorm layer (Norm) and then use a 1×1 convolutional layer ($C_{1\times 1}$) to mix the intra-band dependencies therein, and the input features obtained are described as:

(3) $$\left(I^{h},I^{v}\right)=C_{1\times 1}(Norm(I)),$$

where $I^{v}$ and $I^{h}\in R^{H}{}^{\times W\times D}$ are the input features of intra-SA-V and intra-SA-H, respectively, where D=C/2. For the intra-vertical bars, note that we partition $I^{v}$ into V non-overlapping vertical bars $I_{m}^{v}\in R^{H\times D},m=\left\{1,2,\ldots ,V\right\}$. Each $I_{m}^{v}$ carries H D-dimensional tokens. A vertical strip of the multi-headed participation feature $O_{mn}^{v}\in R^{H\times \frac{D}{s}}$ is described as:

(4) $$O_{mn}^{v}=I_{Softmax}^{v}\left(\frac{Q_{mn}^{v}{\left(K_{mn}^{v}\right)}^{T}}{\sqrt{D/s}}\right)V_{mn}^{v},$$

where $Q_{mn}^{v},K_{mn}^{v},V_{mn}^{v}\in R^{H\times \frac{D}{s}}$ are the query, key, and value of $I_{m}^{v}$ mapping, respectively, and the correspondence between *Q*, *K*, and *V* and the linear projection matrix $P_{n}^{Q},P_{n}^{K},P_{n}^{V}\in R^{D\times \frac{D}{s}},n\in \left\{1,\ldots ,s\right\}$ (the number of times *s* = 5 for setting the frontal in this paper) is $\left(Q_{mn}^{v},K_{mn}^{v},V_{mn}^{v}\right)=\left(I_{m}^{v}P_{n}^{Q},I_{m}^{v}P_{n}^{K},I_{m}^{v}P_{n}^{V}\right)$. Its space complexity is $\sigma \left(H^{2}\right)$.

We stitch the multi-headed vertical feature $O_{mn}^{v}\in R^{H\times \frac{D}{s}}$ along the channel dimension to obtain $O_{m}^{v}\in R^{H\times D}$ and collapse it into a three-dimensional tensor $O^{v}\in R^{H\times W\times D}$ as the intra-SA-V output. Correspondingly, attention is paid within the horizontal strips to the generation of multi-headed participation features corresponding to each horizontal strip, denoted as $O_{mn}^{h}\in R^{W\times \frac{D}{s}}$, whose spatial complexity is $\sigma \left(W^{2}\right)$. Similarly, the intra-SA-H output can be expressed as $O^{h}\in R^{H\times W\times D}$. They are then concatenated and input to a 1×1 convolutional layer with residuals connected to the original input features I. The resulting participating features $O_{S-MSA}\in R^{H\times W\times C}$ are described as:

(5) $$O_{S-MSA}=C_{1\times 1}\left(Concate\left(O^{v},O^{h}\right)\right)+I.$$

where Concate(…) represents the splicing operation. The DGB module shown in Figure 1 is then applied to $O_{S-MSA}$. Specifically, we first generate the final output $O_{\mathrm{intra}}\in R^{H\times W\times C}$ using LayerNorm, a dual gating feedforward network (DGFN) with residual connections, and a 3 × 3 deep convolutional layer conditional positional encoding [8] (CPE) with residual connections. The process is described as:

(6) $$O_{\mathrm{intra}}=CPE\left(DG\left(Norm\left(O_{S-MSA}\right)\right)+O_{S-MSA}\right).$$

The total space complexity of intra-SA is $\sigma \left(WH^{2}+HW^{2}\right)$.

#### 3.3.2. Inter-SA Block

As shown in Figure 4b, an inter-SA block is also divided into two independent features along the channel dimension, vertical inter-band attention (Inter-SA-V) and horizontal inter-band attention (Inter-SA-H), and then they are subjected to SA operations separately. Inter-SA is a mutual concern between strips, considering each strip as a whole characteristic. Similar to intra-SA, we process the input using Equation (3) to generate the input features $I^{v}$ and $I^{h}\in R^{H\times W\times D}$ for inter-SA-V and inter-SA-H, respectively.

For the vertical inter-strip attention, we also generate multi-headed queries, keys, and values via linear projection matrices, analogous to intra-SA, which we simply denote as $Q_{n}^{v},K_{n}^{v},V_{n}^{v}\in R^{H\times W\times \frac{D}{s}}$. Next, we reshape $Q_{n}^{v}$, $K_{n}^{v}$, and $V_{n}^{v}$ into a two-dimensional tensor of size $W\times \frac{D^{v}}{s}$, where $D^{v}=H\times D$, denoting *W* vertical bars of size $\frac{D^{v}}{s}$. Then, the output feature $O_{n}^{v}\in R^{W\times \frac{D^{v}}{s}}$ is described as:

(7) $$O_{n}^{v}=I_{Softmax}^{v}\left(\frac{Q_{n}^{v}{\left(K_{n}^{v}\right)}^{T}}{\sqrt{D^{v}/s}}\right)V_{n}^{v}.$$

Its spatial complexity is $\sigma \left(W^{2}\right)$. Symmetrically, horizontal inter-strip attention generates multi-headed participation features $O_{n}^{h}\in R^{H\times \frac{D^{h}}{s}}$, where $D^{h}=W\times D$, whose spatial complexity in the attention mechanism is $\sigma \left(H^{2}\right)$.

Finally, we splice the multi-head vertical and horizontal features into $O^{v}\in R^{W\times D^{v}}$ and H×W×D along the channel dimension and reshape them into a 3D tensor of size H×W×D. Similar to the intra-SA in Equations (5) and (6), we can generate the final output $O_{\mathrm{int}\mathrm{er}}\in R^{H\times W\times C}$ of the inter-SA block. The total space complexity of inter-SA is $\sigma \left(H^{2}+W^{2}\right)$.

The space complexity of the vanilla transformer can be as high as $\sigma \left(W^{2}H^{2}\right)$. In contrast, our ESIDformer is more efficient, and requires only $\sigma \left(W^{2}H+WH^{2}\right)$. In addition, the designed ESIDformer architecture based on the transformer model combines vertical and horizontal multi-headed attention mechanisms intra-SA and inter-SA to efficiently explore fuzzy directions and capture different degrees of ambiguity. By stacking these interleaved intra-SA and inter-SA blocks, the degree of blurring can be explored in more detail. This improved deblurring method not only requires less memory, but also yields better performance compared to other methods.

### 3.4. Dual Gating Feedforward Network

According to [16,36], Feedforward Networks (FFN) have been found to have limitations in capturing local context and operate only on a single and repetitive basis per pixel. In order to integrate contextual information more effectively during feature transformation, we introduce a dual gating mechanism and local feature enhancement in the FFN architecture, resulting in a new dual-gating feedforward network (DGFN). As shown in Figure 4c, the dual-gate mechanism involves the application of the dual Gaussian error linear unit (GELU) activation function and element-wise product to filter out the less informative features from the two parallel paths, and then the combination of them using element-wise sum to retain the most sensitive information. In addition, 1×1 convolution ($C_{1\times 1}$) and 3 × 3 depth convolution ($C_{3\times 3}$) are used to enrich the local features on each path. When the input size is $F_{i}\in R^{H\times W\times C}$, the complete DGFN can be expressed as:

(8) $$\begin{array}{l} F_{DG}=\varphi \left(C_{3\times 3}^{}C_{1\times 1}^{}F_{i}\right)⊙\left(C_{3\times 3}^{2}C_{1\times 1}^{2}F_{i}\right)\\ +\left(C_{3\times 3}^{}C_{1\times 1}^{}F_{i}\right)⊙\varphi \left(C_{3\times 3}^{2}C_{1\times 1}^{2}F_{i}\right)\\ F_{o}=W_{1\times 1}F_{DG}(F_{i})+F_{i} \end{array},$$

where $F_{o}\in R^{H\times W\times C}$ denotes the output feature, $F_{DG}$ denotes the dual gating mechanism, ⊙ is the element multiplication operation, and φ is the GELU activation function.

### 3.5. Loss Function

The common loss function used for training networks in image recovery tasks is the Mean Square Error (MSE) loss function. MSE calculates the difference between the network output image and the real image at the corresponding pixel points and squares them, but since the squaring operation usually penalizes larger error values and tolerates smaller ones, it leads to over-smoothed output results and blurred image edges. Therefore, this paper is inspired by [46], and uses the self-supervised technique of contrast learning [47] in training the network. It allows the model to generate generic features from similarities and dissimilarities of the data, even without labels. We used contrast learning to make a deblurred output image similar to its ground truth, but different from its blurred input. The contrast loss formula is:

(9) $$L_{\mathrm{con}}=\frac{L_{1}(\zeta (S)-\zeta (R))}{L_{1}(\zeta (X)-\zeta (R))},$$

where X is the fuzzy input, R is the defuzzification result, and S is the associated ground truth, where $X,R,S\in R^{H\times W\times 3}$. We regard X, R, and S as the negative, anchor, and positive samples. ζ denotes the output features of the VGG19 pre-trained network on ImageNet, and the deep convolutional layer before the third pooling layer and after the second convolutional layer is selected as the feature layer to extract hidden features. One of them, VGG19, is a deep convolutional neural network model, developed by a team of researchers at the University of Oxford. ImageNet is a large-scale image database containing millions of labeled high-resolution images. $L_{1}$ denotes the $L_{1}$ parametric number, $L_{1}$ loss does not over-penalize larger error values and is able to preserve image structure and edge information, and the $L_{1}$ loss mathematical expression is

(10) $$L_{1}={\left\|X-Y\right\|}_{1}.$$

The loss function of ESIDformer deblurring is

(11) $$L=L_{\mathrm{char}}+\lambda_{1}L_{\mathrm{edge}}+\lambda_{2}L_{\mathrm{con}},$$

where $L_{char}$ and $L_{edge}$ have the same Charbonnier loss and edge loss as MPRNet [31], and $L_{con}$ is the contrast loss. In this paper, we set to $\lambda_{1}=0.05$, as set in [31], and $\lambda_{2}=0.0005$.

## 4. Experiments

In this section, we evaluate the feasibility of the ESIDformer. Firstly, we described the implementation details of the chosen dataset and network framework. The results of our experiments were then analyzed quantitatively and qualitatively to illustrate the excellence of the model’s generalization ability and performance. Finally, we performed ablation experiments to demonstrate the validity of the design.

### 4.1. Datasets

In order to evaluate blurred images as close to real scenes as possible, this paper first evaluates them on the widely used GoPro dataset [12], which consists of a total of 3214 pairs of blurred and clear images with 720 × 1280 resolution, of which 2103 pairs of blurred and clear images are used as the training set and the remaining 1111 pairs are used as the test set. To verify the generalization ability of the algorithm in this paper, the HIDE dataset [48] is also added for testing. The HIDE dataset covers a wide range of scenes and a variety of motion types, and only 2025 of these images are used for testing in this paper. Finally, to demonstrate the model’s ability to generalize in the real world, we use the RealBlur dataset [49]. One subset of the RealBlur dataset, RealBlur-R, consists of camera-originated images, and the other subset, RealBlur-J, consists of camera-processed JPEG images. The image pairs of the RealBlur dataset were captured in real environments, mainly in ultra-low light and various unnatural light conditions, and contain 4738 image pairs from 232 different scenes, which we evaluated with 980 pairs.

### 4.2. Experimental Environment and Implementation Details

The deep learning framework used for the experiments was PyTorch 1.9.0, the computer operating system was Microsoft Windows 10, and the graphics card model was NVIDIA TITAN XP (12 GB). We trained our network model on the GoPro dataset only. The Adam optimizer with momentum decay exponents β1 = 0.9 and β2 = 0.999 was used to update the network parameters, and the number of training rounds was set to 3000 epochs. The initial learning rate was set to 10^−4^, and the learning rate was reduced and stabilized to 10^−7^ by the cosine annealing strategy. During each round of training iterations, four (Batch size = 4) images cropped to 256 × 256 size were randomly selected as network inputs, the data were enhanced by on-the-fly cropping, random rotation and vertical flipping, and the pixel values of the trained blurred–clear image pairs were normalized to values in the range of [−1, 1] to make the network easier to train. In the paper, the trained models were tested on the GoPro dataset, the HIDE dataset, and the real dataset RealBlur.

### 4.3. Experimental Results

#### 4.3.1. Quantitative Analysis

We compared the trained model with the current mainstream and advanced algorithms [11,14,15,25,30,31,32]. As shown in Table 1, except for the image processing transformer (IPT) [34], which is also a network model built based on transformer architecture as a reference, all of them are defuzzified network models built based on CNN architecture. As can be seen from Table 1, we used Peak Signal-to-Noise Ratio (PSNR) and Structural Similarity (SSIM) as the main evaluation indexes to quantitatively evaluate the recovered image quality, and the proposed ESIDformer in this paper achieved the best results. Compared with other CNN-based traditional methods, our approach does not require a large amount of training data for the generalization of the model. IPT is an efficient transformer architecture that needs to be pre-trained using large amounts of image data and fine-tuned based on the pre-trained model. In contrast, our model was trained only on the GoPro dataset and did not rely on loop structure. It utilized a cross-layer feature fusion block (CFFB) to combine shallow features more efficiently, effectively interacted with local and global information via S-MSA to capture the size and orientation information of ambiguous regions, and utilized a dual-gated feed-forward network (DGFN) to further capture the correlation between local contexts near and far. This approach allowed our model to run efficiently and produce high-quality results. In addition to this, Table 2 and Table 3 evaluated the results for the HIDE and RealBlur datasets, respectively. As can be seen from Table 2 and Table 3, the deblurring effect of ESIDformer for a wide range of scenes and the real world is optimal compared to other mainstream and advanced models.

#### 4.3.2. Qualitative Analysis

**Figure 5:**
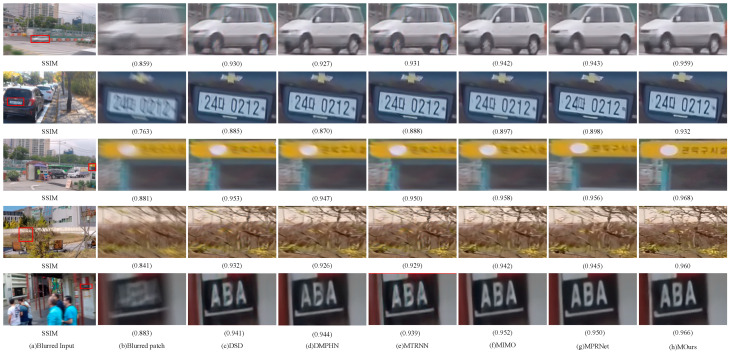
Qualitative comparison of the GoPro dataset (zoomed in for a better view): (**a**) blurred input, (**b**) blurred patch, (**c**) DSD, (**d**) DMPHN, (**e**) MTRNN, (**f**) MIMO, (**g**) MPRNet, and (**h**) ours.

**Figure 6:**
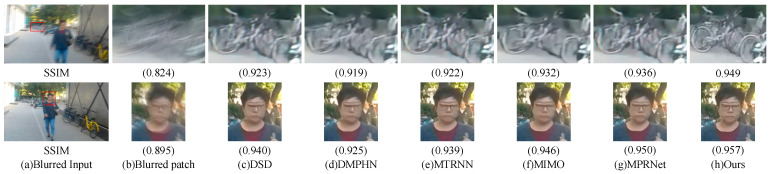
Qualitative comparison of the HIDE dataset (zoomed in for a better view): (**a**) blurred input, (**b**) blurred patch, (**c**) DSD, (**d**) DMPHN, (**e**) MTRNN, (**f**) MIMO, (**g**) MPRNet, and (**h**) ours.

**Figure 7:**
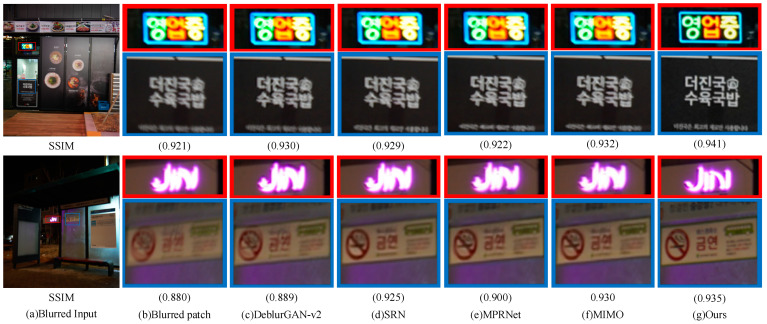
Qualitative comparison of the RealBlur dataset (zoomed in for a better view): (**a**) blurred input, (**b**) blurred patch, (**c**) DeblurGAN-v2, (**d**) SRN, (**e**) MPRNet, (**f**) MIMO, and (**g**) ours.

In addition to the quantitative analysis of the algorithms in the paper through evaluating the metrics PSNR and SSIM, a qualitative visual effect comparison analysis of randomly selected images of different scenes from the GoPro dataset, HIDE dataset, RealBlur-J and RealBlur-R datasets with the current mainstream algorithms was also conducted. Figure 5, Figure 6 and Figure 7 show the visual results of different algorithms for deblurring images on the GoPro dataset, the HIDE dataset, and the RealBlur dataset, respectively. As can be seen in Figure 5, our model can better reconstruct the symbols and numbers in the license plate, the text on the roadside, and the clearer contours of the objects, with a more detailed recovery of the edge texture of the local objects in the image. As can be seen in Figure 6, our model better recovers the features and facial expressions of people in the close view in the HIDE dataset, as well as the contour lines and spatial locations of objects in the far view. As can be seen in Figure 7, in the RealBlur dataset, our model is better able to capture the motion blur produced by text under realistic environmental conditions of low and unnatural light, and can recover sharper results. Overall, the qualitative results show that our model can better capture motion-blurred regions in different environments and obtain better deblurring effects.

The results of the quantitative comparative analysis of each method in Table 1, Table 2 and Table 3, as well as the comparative analysis of the subjective visual effects in Figure 5, Figure 6 and Figure 7, show that the method in this paper can handle non-uniform blur well, reconstruct information such as image edge contours and details better, and deblur more thoroughly. At the same time, compared with the current mainstream and advanced deblurring methods, the method in this paper achieved the best results on the benchmark dataset GoPro, HIDE, and the real dataset RealBlur, with better generalization ability and robustness.

### 4.4. Ablation Studies

In order to verify the effectiveness of the designed modules in the improvement of the network deblurring performance, this paper trained and tested the computational analysis of different modules on the evaluation metrics on the GoPro dataset and evaluated them using the ablation of each module added in turn. We will analyze the impact of each module on the final performance of the network in terms of the designed and improved stripe-based multi-head self-attention (S-MSA), dual-gating feedforward network (DGFN), and cross-layer feature fusion block (CFFB). For the sake of experimental fairness, we train on the proposed ESIDformer network, a hybrid residual encoder–decoder architecture consisting of CNN and a transformer.

#### 4.4.1. Stripe-Based Multi-Head Self-Attention

**Figure 8:**
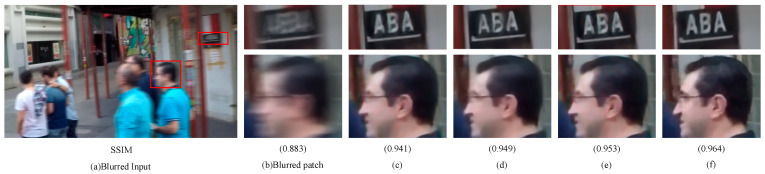
Visualization of the impact of network architecture and individual modules on GoPro (zoomed in for a better view): (**a**) blurred input, (**b**) blurred patch, (**c**) w/o A DGFN, (**d**) w/o B DGFN, (**e**) w/o CFFB, and (**f**) ours. W/o stands for without. In (**c**,**d**), w/o A represents the DGFN without deep convolution and w/o B represents the DGFN without the double gating mechanism.

As can be seen from Table 4, the ablation experiments were performed based on S-MSA. S-MSA collaborates intra- and inter-band attention blocks in vertical and horizontal directions to solve the problem of blurred regions and blurred pattern search, and makes good use of the original image information to achieve good deblurring. As shown in Figure 8, S-MSA was able to recover the local and global information of text and face in the image better.

#### 4.4.2. Dual-Gating Feed-Forward Network

The addition of the DGFN module makes the feature transformation efficient, again emphasizing the contextual linkage and yielding better performance. In this case, a separate ablation analysis was carried out for the DGFN added to the transformer block, which is integrated together in Table 4. Meanwhile, combining the quantitative analysis of Table 4 with the qualitative analysis of Figure 8 shows the importance of the gating mechanism for performing controlled feature transformations and the small gain that the introduction of deep convolution brings to the capture of local information.

#### 4.4.3. Cross-Layer Feature Fusion Block

The addition of the CFFB module provides an effective aid to the exchange of feature information between layers in the process of feature extraction and image deblurring. In this section, the CFFB modules in the network are added and removed as a whole, and individual CFFB modules are not discussed separately. As can be seen from Table 4 and Figure 8, there is a gain in improving the deblurring performance.

## 5. Conclusions

In this paper, we propose an efficient stripe image deblurring network based on a hybrid architecture of CNN and a transformer, which can better fuse and interact with local and global information of images so as to efficiently and effectively recover information such as image edge contours and texture details. In particular, we propose a cross-layer attention fusion module that improves the efficiency of using parameters and emphasizes the exchange of information between different scales. Secondly, we improve the stripe-based transformer block using multi-headed self-attentiveness based on intra- and inter-stripe, and improve and propose a dual-gating feedforward network that allows computational resources of different sizes and orientations in the image to be more rationally allocated, while reducing the loss of high-frequency detail information during network transmission. Finally, the cross-layer feature fusion module is again used for the complementary integration of information. The objective evaluation and subjective visual results are better than the current advanced deblurring algorithms on the benchmark datasets GoPro and HIDE, as well as the real dataset RealBlur.

## Figures and Tables

**Table 1:** Quantitative ablation study on GoPro (↑ higher is better, ↓ lower is better).

Method	PSNR (M_PSRN_) ↑	SSIM (M_SSIM_) ↑	Params (M) ↓	σ_PSRN_/σ_SSIM_
DeblurGAN-v2	29.08	0.918	68	-
SRN	30.24	0.934	7	-
DSD	30.96	0.942	3	-
MTRNN	31.12	0.944	3	-
DMPHN	31.39	0.947	22	-
RADN †	31.85	0.953	-	-
SAPHN †	32.02	0.953	-	-
MIMO	32.45	0.957	16	-
MPRNet	32.65	0.958	20	-
IPT †	32.52	-	114	-
Ours	**33.11 (33.1182)**	**0.963 (0.9632)**	32	0.0040/0.0023

Here, † denotes the weight of unpublished code or pretraining for this work. The best scores in each column are labeled in bold. Params are calculated in units of (M). M_PSRN_ and M_SSIM_ represent the average performance and σ_PSRN_/σ_SSIM_ represents the value of standard deviation, respectively.

**Table 2:** Quantitative ablation study on HIDE (↑ higher is better, ↓ lower is better).

Method	PSNR (M_PSRN_) ↑	SSIM (M_SSIM_) ↑	Params(M) ↓	σ_PSRN_/σ_SSIM_
DeblurGAN-v2	27.51	0.884	68	-
SRN	28.36	0.903	7	-
DSD	29.01	0.913	3	-
DMPHN	29.11	0.917	22	-
MTRNN	29.15	0.917	22	-
MIMO	30.00	0.930	16	-
MPRNet	30.96	0.939	20	-
Ours	**31.10 (31.09176)**	**0.948 (0.9482)**	32	0.1456/0.0010

Params are calculated in units of (M). M_PSRN_ and M_SSIM_ represent the average performance and σ_PSRN_/σ_SSIM_ represents the value of standard deviation, respectively.

**Table 3:** Quantitative ablation study on RealBlur (↑ higher is better, ↓ lower is better).

Model	RealBlur-J		RealBlur	σ_PSRN_/σ_SSIM_
	PSNR (M_PSRN_) ↑	SSIM (M_SSIM_) ↑	Params(M) ↓	
DeblurGANv2	29.69	0.870	68	-
SRN	31.38	0.909	7	-
MPRNet	31.76	0.922	20	-
SPAIR †	31.82	0.922	-	-
MIMO	31.92	0.919	16	-
Ours	**32.50 (32.5216)**	**0.931 (0.9309)**	32	0.2845/0.0012

Here, † denotes the weight of unpublished code or pretraining for this work. Params are calculated in units of (M). M_PSRN_ and M_SSIM_ represent the average performance and σ_PSRN_/σ_SSIM_ represents the value of standard deviation, respectively.

**Table 4:** Performance of each module on the GoPro dataset.

S-MSA	DGFN (w/o A)	DGFN (w/o B)	CFFB	PSNR
√				32.89
√	√			32.94
√		√		33.07
√	√	√	√	33.11

## Data Availability

Public datasets.
